# Assessment of heterogeneity according to hospital or medical experience factors in outcomes of chemotherapy for advanced biliary tract cancer: a post-hoc analysis of JCOG1113

**DOI:** 10.1093/jjco/hyae188

**Published:** 2025-01-08

**Authors:** Koh Fukushi, Hiroshi Imaoka, Masafumi Ikeda, Junki Mizusawa, Chigusa Morizane, Takuji Okusaka, Satoshi Kobayashi, Naoki Sasahira, Satoshi Shimizu, Kentaro Yamazaki, Naohiro Okano, Haruo Miwa, Kazuo Hara, Sohei Satoi, Keiji Sano, Kenji Sakai, Rie Sugimoto, Kazuyoshi Nakamura, Takeshi Terashima, Masato Ozaka, Makoto Ueno, JCOG Hepatobiliary and Pancreatic Oncology Group, Koh Fukushi, Hiroshi Imaoka, Masafumi Ikeda, Chigusa Morizane, Takuji Okusaka, Satoshi Kobayashi, Naoki Sasahira, Satoshi Shimizu, Kentaro Yamazaki, Naohiro Okano, Haruo Miwa, Kazuo Hara, Sohei Satoi, Keiji Sano, Kenji Sakai, Rie Sugimoto, Kazuyoshi Nakamura, Takeshi Terashima, Masato Ozaka, Makoto Ueno, Junki Mizusawa

**Affiliations:** Department of Hepatobiliary and Pancreatic Oncology, National Cancer Center Hospital East, Kashiwa, Japan; Department of Hepatobiliary and Pancreatic Oncology, National Cancer Center Hospital East, Kashiwa, Japan; Department of Hepatobiliary and Pancreatic Oncology, National Cancer Center Hospital East, Kashiwa, Japan; Japan Clinical Oncology Group Data Center, Clinical Research Support Office, National Cancer Center Hospital, Tokyo; Department of Hepatobiliary and Pancreatic Oncology, National Cancer Center Hospital, Tokyo, Japan; Department of Hepatobiliary and Pancreatic Oncology, National Cancer Center Hospital, Tokyo, Japan; Department of Gastroenterology, Kanagawa Cancer Center, Yokohama, Japan; Department of Hepato-Biliary-Pancreatic Medicine, Cancer Institute Hospital, Japanese Foundation for Cancer Research, Tokyo, Japan; Department of Gastroenterology, Saitama Cancer Center, Saitama, Japan; Division of Gastrointestinal Oncology, Shizuoka Cancer Center, Shizuoka, Japan; Department of Medical Oncology, Kyorin University Faculty of Medicine, Tokyo, Japan; Gastroenterological Center, Yokohama City University Medical Center, Yokohama, Japan; Department of Gastroenterology, Aichi Cancer Center Hospital, Nagoya, Japan; Department of Pancreatobiliary Surgery, Kansai Medical University, Hirakata, Japan; Department of Surgery, Teikyo University School of Medicine, Tokyo, Japan; Department of Gastroenterology and Hepatology, NHO Osaka National Hospital, Osaka, Japan; Department of Hepato-Biliary-Pancreatology, NHO Kyushu Cancer Center, Fukuoka, Japan; Division of Gastroenterology, Chiba Cancer Center, Chiba, Japan; Department of Gastroenterology, Kanazawa University Hospital, Kanazawa, Japan; Department of Hepato-Biliary-Pancreatic Medicine, Cancer Institute Hospital, Japanese Foundation for Cancer Research, Tokyo, Japan; Department of Gastroenterology, Kanagawa Cancer Center, Yokohama, Japan

**Keywords:** biliary tract cancer, inter-institutional heterogeneity, chemotherapy, hospital volume, biliary intervention

## Abstract

**Background:**

JCOG1113 is a randomized phase III trial that showed non-inferiority of gemcitabine plus S-1 to gemcitabine plus cisplatin in patients with advanced biliary tract cancer. Assessment of inter-institutional heterogeneity in chemotherapy contributes to confirm generalizability and reliability of the study itself. However, there have been no studies conducted to assess the heterogeneity among participating centers in randomized phase III trials for biliary tract cancer.

**Methods:**

The objective of this post-hoc analysis was to assess the inter-institutional heterogeneity in the overall survival and progression-free survival of patients with advanced biliary tract cancer treated with first-line chemotherapy in the JCOG1113 trial. The heterogeneity in the overall survival and progression-free survival was assessed according to three factors: hospital volume, experience in medical oncology and experience in biliary intervention. A total of 300 advanced biliary tract cancer patients were analyzed. There were no statistically significant trends observed between hospital volume, experience in medical oncology, or experience in biliary intervention and overall survival (hospital volume: adjusted trend *P* value = 0.6796; experience in medical oncology: adjusted trend *P* value = 0.4092; experience in biliary intervention: adjusted trend *P* value = 0.6112). Similarly, no statistically significant trends were observed between these factors and progression-free survival (hospital volume: adjusted trend *P* value = 0.3000; experience in medical oncology: adjusted trend *P* value = 0.1108; experience in biliary intervention: adjusted trend *P* value = 0.2898).

**Conclusions:**

This study revealed no inter-institutional heterogeneity in the overall survival and progression-free survival in the JCOG1113 study population of advanced biliary tract cancer patients.

## Teaser

Our study revealed no significant inter-institutional heterogeneity of the survival outcomes in advanced biliary tract cancer patients of the JCOG1113 study, supporting the generalizability of its results.

## Introduction

Biliary tract cancer (BTC) is a relatively rare cancer that arises from the intrahepatic or extrahepatic bile ducts or gallbladder. In advanced cases (recurrent or unresectable), systemic chemotherapy is the standard treatment, but the prognosis is generally poor. Gemcitabine plus cisplatin (GC) has long been the first-line treatment for advanced cases, but the JCOG1113 trial, a randomized phase III trial revealed non-inferiority of gemcitabine plus S-1 (GS) to GC [1].

Various factors can affect the clinical outcomes of chemotherapy in patients with advanced cancers. Among the well-recognized factors are patient-related factors, such as the performance status and presence of comorbidities and inflammation-related factors, as assessed by serum markers such as albumin and C-reactive protein [2]. On the other hand, the importance of ancillary medical factors is often overlooked, such as the facilities available at the treating hospital (hospital facilities) and experience of each facility and each oncologist. It has been reported that in patients with unresectable pancreatic cancer, oncologists with more experience showed a higher number of transitions to chemotherapy and a longer survival rate [3]. However, no studies until date have been conducted to evaluate inter-institutional heterogeneity in the outcomes of chemotherapy in patients with advanced BTC. Furthermore, patients with BTC often present with jaundice and cholangitis, which are potentially fatal in these patients [4]. In such situations, biliary drainage is required both before and during chemotherapy. However, the two mainly used drainage techniques, namely, percutaneous biliary drainage and endoscopic biliary drainage, require highly specialized skills [5]. In addition, biliary drainage strategies vary among institutions and physicians. High volume centers may have higher endoscopic retrograde cholangiopancreatography (ERCP) success rates and lower adverse event (AE) rates [6,7], even for malignant biliary strictures [8]. Most importantly, these factors may have an impact on the survival of BTC patients. Thus, we speculate that various factors interact to affect overall survival (OS) in BTC and inter-institutional heterogeneity in the outcomes of chemotherapy for BTC may be greater than that for other cancer types. The purpose of this study was to investigate the inter-institutional heterogeneity in the survival outcomes of advanced BTC patients treated by chemotherapy in the JCOG1113 trial, according to the hospital volume, experience in medical oncology and experience in biliary intervention.

## Method

### Study design

Data for this post-hoc analysis were derived from the JCOG1113, a phase III trial comparing GC and GS in patients with advanced BTC. Details of the study design, the inclusion/exclusion criteria and the safety/efficacy results of the trial have been described previously [1]. Briefly, the primary endpoint of the trial was the OS. The study protocol of JCOG1113 was approved by the ethics committee or the Institutional Review Board (IRB) of each participating center. Written informed consent, including secondary use of data, was obtained from each patient before enrollment in JCOG1113.

In this post-hoc analysis, inter-institutional heterogeneity between 2013 and 2015 was evaluated based on three key factors: (i) Hospital volume—higher-volume centers typically provide enhanced patient care through comprehensive services, including specialized nutritional management, interdisciplinary collaboration with pharmacists and nurses and advanced supportive care. These elements are crucial for optimal management of BTC patients and may influence their survival outcomes. (ii) Experience in medical oncology—the level of experience in medical oncology directly impacts the effectiveness of chemotherapeutic interventions for BTC patients. (iii) Experience in biliary intervention—proficiency in biliary interventions is essential for managing potentially life-threatening complications such as jaundice and cholangitis, which are common in BTC patients.

We calculated sample sizes for the following factors from January 2013 to December 2015 and categorized them into three categories at the tertile points: low, medium and high.

Hospital volume was defined as the number calculated by multiplying the total number of patients who started first-line chemotherapy for advanced BTC patients by the total number of medical oncologists who performed pancreaticobiliary chemotherapy. Experience in medical oncology was defined as the average years of experience as medical oncologists for doctors who participated as co-investigators in JCOG 1113. Experience in biliary intervention was defined as the total number of all biliary interventions, including endoscopic and percutaneous drainage.

We sent questionnaires to JCOG1113 participating institutions. The questionnaires enquired three queries between January 2013 and December 2015: cumulative number of BTC patients receiving first-line chemotherapy, mean years of experience as a medical oncologist participating in JCOG1113 and total number of endoscopic or percutaneous biliary drainage. However, hospitals with <2 BTC patients enrollments in either the GC or GS arms (14 hospitals) and three institutions without response to our questionnaires were excluded from the assessment. All of the three factors were categorized into tertiles (lowest, intermediate and highest tertile).

### Objectives

The primary objective of this post-hoc analysis was to assess the inter-institutional heterogeneity of the OS in advanced BTC patients treated with first line chemotherapy. The secondary objective was to assess the inter-institutional heterogeneity in the progression-free survival (PFS) in the same study population.

### Statistical analysis

We tested the hypothesis that favorable ancillary institutional factors are associated with longer survival in advanced BTC patients receiving chemotherapy. Univariable analysis was performed by the chi-square test for categorical variables, and the Kruskal–Wallis test for continuous variables. OS was defined as the period from the date of randomization to the date of death or censored on the last date on which a patient was known to be alive. PFS was defined as the period from the date of randomization and to the date of first documentation of disease progression or date of death from any cause. Tumor response was assessed according to the Response Evaluation Criteria in Solid Tumors version 1.1. If there was no documentation of disease progression or death by the data cutoff time, the PFS was censored at the last adequate tumor assessment. The HRs for OS, those for PFS and trend *P* values across three groups were estimated using the Cox proportional hazards model, with adjustments for sex distribution, age, distribution of the Eastern Cooperative Oncology Group performance status (ECOG PS), distribution of disease stage, distribution of primary site and need for biliary drainage. *P* values among three groups were calculated using the log-rank test or Cox regression analysis. *P* values of <0.05 were considered as denoting statistical significance and all *P* values were two-sided. Data were analyzed using SAS 9.4 (SAS Institute Inc., Cary, NC, USA).

## Results

### Patient characteristics

**Figure 1:**
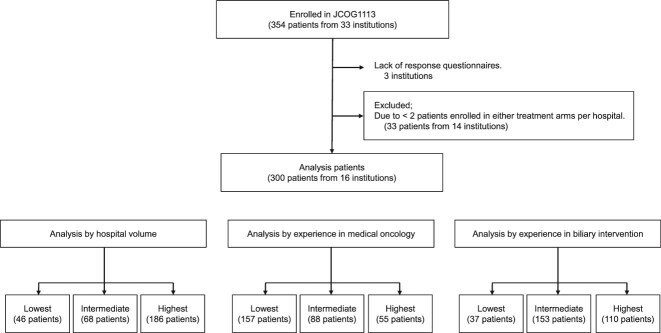
Study flow chart.

**Figure 2:**
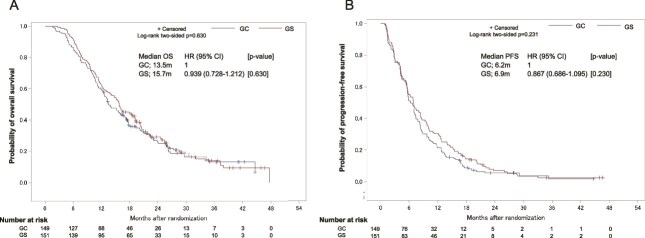
Kaplan–Meier curves for both overall survival (OS) (A) and progression-free survival (PFS) (B) in eligible advanced biliary tract cancer (BTC) patients treated with GS or GC.

The patient flow chart is shown in Fig. 1. Of the 33 institutions that participated in the JCOG1113 study, 14 were excluded from this analysis since <2 patients were enrolled in either the GC or GS arms from these institutions. In addition, three more institutions were excluded because they failed to provide responses to our questionnaire. Consequently, of the 354 patients enrolled in JCOG1113, 300 patients (including 149 in the GC arm and 151 in the GS arm) from 16 institutions were analyzed. The patient characteristics are summarized in Table 1. The Kaplan–Meier curves for determining the OS and PFS in the eligible patients treated with GC or GS are shown in Fig. 2. The median OS was 13.5 months in the GC arm and 15.7 months in the GS arm (HR, 0.939; 95% confidence interval [CI], 0.728–1.212). The median PFS was 6.2 months in the GC arm and 6.9 months in the GS arm (HR, 0.867; 95% CI, 0.686–1.095).

**Table 1:** Patient characteristics

	GC	GS	Total
	*n* = 149	*n* = 151	*N* = 300
Age, years			
Median	67	68	67
(Range)	(45–78)	(27–79)	(27–79)
Sex			
Male	87 (58.4%)	83 (55.0%)	170
Female	62 (41.6%)	68 (45.0%)	130
ECOG PS			
0	107 (71.8%)	104 (68.9%)	211
1	42 (28.2%)	47 (31.1%)	89
Disease stage			
Localized	23 (15.4%)	29 (19.2%)	52
Metastatic	89 (59.7%)	87 (57.6%)	176
Recurrent	37 (24.8%)	35 (23.2%)	72
Primary site			
Gallbladder	60 (40.3%)	58 (38.4%)	118
Intrahepatic	43 (28.9%)	36 (23.8%)	79
Extrahepatic	41 (27.5%)	52 (34.4%)	93
*-hilar*	22	30	52
*-distal*	19	22	41
Ampulla of Vater	5 (3.4%)	5 (3.3%)	10
Biliary intervention			
No	90 (60.4%)	85 (56.3%)	175
Yes	59 (39.6%)	66 (43.7%)	125
Prior primary resection			
No	112 (75.2%)	116 (76.8%)	228
Yes	37 (24.8%)	35 (23.2%)	72

ECOG PS, Eastern Cooperative Oncology Group performance status; GC, gemcitabine plus cisplatin; GS, gemcitabine plus S-1.

### Hospital volume

**Figure 3:**
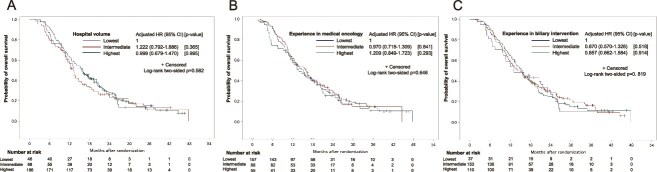
Kaplan–Meier curves for OS according to the hospital volume (A), experience in medical oncology (B) and experience in biliary intervention (C).

In regard to the hospital volume, 46, 68 and 186 patients, respectively, were enrolled from institutions classified into the lowest, intermediate and highest tertiles according to the hospital volume. The characteristics of the patients stratified by the hospital volume are shown in Supplementary Table 1. The Kaplan–Meier curves for OS according to the hospital volume are shown in Fig. 3A. There was no statistical trend between hospital volume and OS (unadjusted trend *P* value = 0.5270, adjusted trend *P* value = 0.6796). The OS did not differ significantly among patients from institutions classified into the highest, intermediate and lowest tertiles by the hospital volume (15.7, 13.0 and 16.0 months, respectively; log-rank *P* = 0.582; adjusted HR (intermediate [vs lowest]) 1.222, 95% CI [0.792–1.886]; (highest [vs lowest]) 0.999, 95% CI [0.679–1.470]). When analyzed by the treatment regimen used, the OS did not differ significantly among patients treated with either GC or GS from institutions classified into the highest, intermediate and lowest tertiles by the hospital volume (Supplementary Table 2).

**Figure 4:**
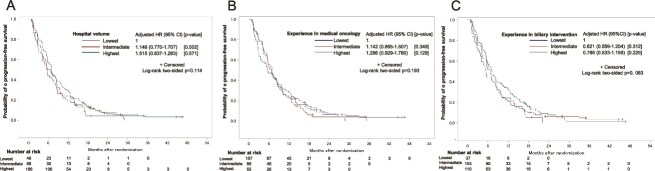
Kaplan–Meier curves for PFS according to the hospital volume (A), experience in medical oncology (B) and experience in biliary intervention (C).

The Kaplan–Meier curves for PFS according to the hospital volume are shown in Fig. 4A. There was no statistical trend between hospital volume and PFS (unadjusted trend *P* value = 0.1160, adjusted trend *P* value = 0.3000). The PFS did not differ significantly among patients from institutions classified into the highest, intermediate and lowest tertiles by hospital volume (6.9, 5.7 and 6.1 months, respectively; log-rank *P* = 0.114; adjusted HR (intermediate (vs lowest)) 1.146, 95% CI [0.770–1.707]; (highest (vs lowest)) 0.904, 95% CI [0.637–1.283]. When analyzed by the treatment regimen used, the PFS did not differ significantly among patients treated with either GC or GS from institutions classified into the highest, intermediate and lowest tertiles by the hospital volume (Supplementary Table 2).

### Experience in medical oncology

In regard to experience in medical oncology, 157, 88 and 55 patients were recruited from institutions classified into the highest, intermediate and lowest tertiles by the experience in medical oncology. The patient characteristics stratified by the experience in medical oncology are shown in Supplementary Table 3.

The Kaplan–Meier curves for OS according to the experience in medical oncology are shown in Fig. 3B. There was no statistical trend between experience in medical oncology and OS (unadjusted trend *P* value = 0.6484, adjusted trend *P* value = 0.4092). The OS did not differ significantly among patients from institutions classified into the highest, intermediate and lowest tertiles by the experience in medical oncology (13.9, 16.0 and 15.1 months, respectively; log-rank *P* = 0.646; adjusted HR (intermediate [vs lowest]) 0.970, 95% CI [0.718–1.309]; (highest [vs lowest]) 1.209, 95% CI [0.849–1.723]. When analyzed by the treatment regimen used, the OS did not differ significantly among patients treated with either GC or GS from institutions classified into the highest, intermediate and lowest tertiles by the experience in medical oncology (Supplementary Table 4).

The Kaplan–Meier curves for PFS according to the experience in medical oncology of the participating institutions are shown in Fig. 4B. There was no statistical trend between experience in medical oncology and PFS (unadjusted trend *P* value = 0.1951, adjusted trend *P* value = 0.1108). The PFS did not differ significantly among patients from institutions classified into the highest, intermediate and lowest tertiles by the experience in medical oncology (5.6, 6.7 and 6.9 months, respectively; log-rank *P* = 0.193; adjusted HR (intermediate [vs lowest]) 1.142, 95% CI [0.865–1.507]; (highest [vs lowest]) 1.286, 95% CI [0.929–1.780]. When analyzed by the treatment regimen used, the PFS did not differ significantly among patients treated with either GC or GS from institutions classified into the highest, intermediate and lowest tertiles by the experience in medical oncology (Supplementary Table 4).

### Experience in biliary intervention

In regard to the experience in biliary intervention at the institutions, 157, 88 and 55 patients were enrolled from institutions classified into the highest, intermediate and lowest tertiles by their experience level in biliary intervention. The patient characteristics stratified by the experience in biliary intervention are shown in Supplementary Table 5.

The Kaplan–Meier curves for OS according to the experience in biliary intervention are shown in Fig. 3C. There was no statistical trend between experience in biliary intervention and OS (unadjusted trend *P* value = 0.8202, adjusted trend *P* value = 0.6112). The OS did not differ significantly among patients from institutions classified into highest, intermediate and lowest tertiles by the experience in biliary intervention (15.5, 15.1 and 13.1 months, respectively; log-rank *P* = 0.819; adjusted HR (intermediate [vs lowest]) 0.870, 95% CI [0.570–1.328]; (highest [vs lowest]) 1.024, 95% CI [0.662–1.548]. When analyzed by the treatment used, the OS did not differ significantly among patients treated either with GC or GS from institutions classified into the highest, intermediate and lowest tertiles by the experience in biliary intervention (Supplementary Table 6).

The Kaplan–Meier curves for PFS according to the experience in biliary intervention are shown in Fig. 4C. There was no statistical trend between experience in biliary intervention and PFS (unadjusted trend *P* value = 0.0842, adjusted trend *P* value = 0.2898). The PFS did not differ significantly among patients from institutions classified into the highest, intermediate and lowest tertiles by the experience in biliary intervention (7.2, 6.8 and 5.5 months, respectively; log-rank *P* = 0.083; adjusted HR (intermediate [vs lowest]) 0.821, 95% CI [0.559–1.204]; (highest [vs lowest]) 0.786, 95% CI [0.533–1.159]. When analyzed by the treatment used, the PFS did not differ significantly among patients treated with either GC or GS from institutions classified into the highest, intermediate and lowest tertiles by the experience in biliary intervention (Supplementary Table 6).

## Discussion

We assessed the inter-institutional heterogeneity of the survival outcomes according to the hospital volume, experience in biliary intervention and medical oncology in advanced BTC patients receiving first-line chemotherapy. The results of this post-hoc analysis indicated that there was no inter-institutional heterogeneity in terms of OS and PFS in advanced BTC patients treated with either GC or GS. This means our study group appears to have maintained institutional quality, with experience not only in medical oncology but also in biliary drainage.

There are several reports on the inter-institutional heterogeneity of the outcomes of chemotherapy for various kinds of cancers. Kawamoto et al. [9] reported inter-institutional heterogeneity in the outcomes of systemic chemotherapy in 8929 pancreatic cancer patients. They reported that the larger hospital volume, the longer OS achieved in patients treated by chemotherapy. Similarly, inter-institutional heterogeneity in the chemotherapy outcomes has been reported for cases of non-small cell lung cancer [10], colorectal cancer [11] and gastric cancer [12]. However, their influence on BTC has also not been well evaluated. BTC is often associated with critical complications and requires highly skilled supportive care, such as biliary drainage, which led to our concern about possible inter-institutional heterogeneity according to the hospital volume, experience in biliary intervention and medical oncology. However, our findings did not support our initial hypothesis.

One possible explanation for these findings is that there were limited treatment options available for patients with advanced BTC. Various guidelines recommend GC-based chemotherapy as a first-line treatment for advanced BTC [4,13], and fluorouracil-based chemotherapy as a second-line treatment [14]. Second explanation is that both GC and GS have low toxicity and regimens are relatively easy to manage. Therefore, these regimens can be easily adopted at various institutions. Third explanation is that the inclusion/exclusion criteria and treatment plan of both GC and GS were predefined by the study protocol. Thus, treatment strategies were standardized across various institutions. Forth explanation is that this study only included the leading institutions in Japan for chemotherapy for advanced BTC that met the mandatory eligibility qualifications and requirements, such as the presence of an IRB and the ability to accept audits.

The JCOG1113 study was conducted based only on the data of patients treated by cytotoxic chemotherapy. However, new combination therapies including immune checkpoint inhibitors combined with cytotoxic agents, such as GC plus durvalumab [15] and GC plus pembrolizumab [16], are emerging as a first-line treatment for advanced BTC. These new regimens could cause specific immune-related AEs that may require specialized treatment. Therefore, the clinical experience of the oncologist may have an influence on the quality of patient management. It is unclear whether the results of this study can be applied to patients receiving immune checkpoint inhibitors. Furthermore genomic therapy is available for BTCs harboring the *FGFR2* fusion gene [17], *BRAF-V600E* mutation [18] and *IDH1* gene mutation [19], and if these are included, a wider range of treatment options will be applicable. However, this study was conducted before such genomic treatments were introduced.

This research had some limitations. First, in the treatment of advanced BTC, in addition to chemotherapy and biliary drainage, various supportive care components [14,20], such as nutritional management, comprehensive care are also essential. However, these components of supportive care were difficult to quantify and could not be included in this study. Second, this study was based on a patient population at facilities affiliated to the JCOG, and the results of this study are based on the same study protocol. Therefore, it is unclear whether the results could be generalized to special target groups such as patients with a poor performance status or elderly patients who fall outside the eligibility criteria. Third, we collected data from the JCOG participating institutions and excluded hospitals with <2 BTC patient enrollments in the GC or GS arms. Therefore, the validity of our results for institutions that did not participate in JCOG1113 or that truly have less experience treating patients with advanced BTC has not been established.

## Conclusions

This study showed the absence of any inter-institutional heterogeneity of the survival outcomes in the advanced BTC patient population of the JCOG1113 study receiving first-line chemotherapy according to the hospital volume, experience in medical oncology, or experience in biliary intervention, indicating that both GC and GS are standardized treatments that can be adopted in various types of institutions.

## Supplementary Material

supple_Table_1_revise_hyae188

supple_Table_2_revise_hyae188

supple_Table_3_revise_hyae188

supple_Table_4_revise_hyae188

supple_Table_5_revise_hyae188

supple_Table_6_revise_hyae188

## Data Availability

The data related to this study cannot be shared publicly due to the need for protection of the privacy of the individual participants of the study. The data will be shared with investigators whose proposed use of the data has been approved by investigators from the JCOG Hepatobiliary and Pancreatic Oncology Group. Proposals should be directed to the corresponding author.

## Notes

**Role of the funder:**

The funder did not play any role in the design of the study; the collection, analysis, or interpretation of the data; the writing of the manuscript; or the decision to submit the manuscript for publication.
