# Supplementary material for: Assessment of heterogeneity according to hospital or medical experience factors in outcomes of chemotherapy for advanced biliary tract cancer: a post-hoc analysis of JCOG1113
Source: Jpn J Clin Oncol. 2025 Jan 8;55(4):355–61. doi: 10.1093/jjco/hyae188 (PMC11973634; doi:10.1093/jjco/hyae188)
Supplement: supple_Table_1_revise_hyae188 [file supple_table_1_revise_hyae188.docx]

Supplemental table 1. Patient characteristics in tertile groups divided by hospital volume.

|  | Lowest  （score;  144-684） | Intermediate（score;  816-2289） | Highest  （score;  3284-4313） | Total | P value |
| --- | --- | --- | --- | --- | --- |
|  | n=46 | n=68 | n=186 | N=300 |  |
| Treatment |  |  |  |  | 0.923 |
| GC | 24 (52.2%) | 34 (50.0%) | 91 (48.9%) | 149 |  |
| GS | 22 (47.8%) | 34 (50.0%) | 95 (51.1%) | 151 |  |
| Age, years |  |  |  |  | 0.657 |
| Median | 68 | 67 | 67.5 | 67 |  |
| (Range) | (45-79) | (35-78) | (27-79) | (27-79) |  |
| Sex |  |  |  |  | 0.193 |
| Male | 30 (65.2%) | 42 (61.8%) | 98 (52.7%) | 170 |  |
| Female | 16 (34.8%) | 26 (38.2%) | 88 (47.3%) | 130 |  |
| ECOG PS |  |  |  |  | 0.626 |
| 0 | 35 (76.1%) | 48 (70.6%) | 128 (68.8%) | 211 |  |
| 1 | 11 (23.9%) | 20 (29.4%) | 58 (31.2%) | 89 |  |
| Disease stage |  |  |  |  | 0.347 |
| Localized | 7 (15.2%) | 8 (11.8%) | 37 (19.9%) | 52 |  |
| Metastatic | 31 (67.4%) | 40 (58.8%) | 105 (56.4%) | 176 |  |
| Recurrent | 8 (17.4%) | 20 (29.4%) | 44 (23.7%) | 72 |  |
| Primary site |  |  |  |  | 0.056 |
| Gallbladder | 23 (50.0%) | 27 (39.7%) | 68 (36.6%) | 118 |  |
| Intrahepatic | 12 (26.1%) | 14 (20.6%) | 53 (28.5%) | 79 |  |
| Extrahepatic | 7 (15.2%) | 25 (36.8%) | 61 (32.8%) | 93 |  |
| *-hilar* | 4 | 10 | 38 | 52 |  |
| *-distal* | 3 | 15 | 23 | 41 |  |
| Ampulla of Vater | 4 (8.7%) | 2 (2.9%) | 4 (2.1%) | 10 |  |
| Biliary drainage |  |  |  |  | 0.184 |
| No | 29 (63.0%) | 45 (66.2%) | 101 (54.3%) | 175 |  |
| Yes | 17 (37.0%) | 23 (33.8%) | 85 (45.7%) | 125 |  |
| Prior primary resection |  |  |  |  | 0.332 |
| No | 38 (82.6%) | 48 (70.6%) | 142 (76.3%) | 228 |  |
| Yes | 8 (17.4%) | 20 (29.4%) | 44 (23.7%) | 72 |  |

ECOG PS, Eastern Cooperative Oncology Group performance status; GC, gemcitabine plus cisplatin; GS, gemcitabine plus S-1.
