# Supplementary material for: Assessment of heterogeneity according to hospital or medical experience factors in outcomes of chemotherapy for advanced biliary tract cancer: a post-hoc analysis of JCOG1113
Source: Jpn J Clin Oncol. 2025 Jan 8;55(4):355–61. doi: 10.1093/jjco/hyae188 (PMC11973634; doi:10.1093/jjco/hyae188)
Supplement: supple_Table_3_revise_hyae188 [file supple_table_3_revise_hyae188.docx]

Supplemental table 3. Patient characteristics in tertile groups divided by experience in medical oncology.

|  | Lowest  （score;  5.7-10.6） | Intermediate  （score;  10.7-13.5） | Highest  （score;  14.9-23.7） | Total | P value |
| --- | --- | --- | --- | --- | --- |
|  | N=157 | N=88 | N=55 | n=300 |  |
| Treatment |  |  |  |  | 0.904 |
| GC | 78 (49.7%) | 45 (51.1%) | 26 (47.3%) | 149 |  |
| GS | 79 (50.3%) | 43 (48.9%) | 29 (52.7%) | 151 |  |
| Age, years |  |  |  |  | 0.501 |
| Median | 68 | 66.5 | 68 | 67 |  |
| (Range) | (27-79) | (45-78) | (35-79) | (27-79) |  |
| Sex |  |  |  |  | 0.053 |
| Male | 86 (54.8%) | 45 (51.1%) | 39 (70.9%) | 170 |  |
| Female | 71 (45.2%) | 43 (48.9%) | 16 (29.1%) | 130 |  |
| ECOG PS |  |  |  |  | 0.312 |
| 0 | 105 (66.9%) | 67 (76.1%) | 39 (70.9%) | 211 |  |
| 1 | 52 (33.1%) | 21 (23.9%) | 16 (29.1%) | 89 |  |
| Disease stage |  |  |  |  | 0.325 |
| Localized | 32 (20.4%) | 12 (13.6%) | 8 (14.6%) | 52 |  |
| Metastatic | 94 (59.9%) | 52 (59.1%) | 30 (54.5%) | 176 |  |
| Recurrent | 31 (19.7%) | 24 (27.3%) | 17 (30.9%) | 72 |  |
| Primary site |  |  |  |  | 0.739 |
| Gallbladder | 62 (39.5%) | 33 (37.5%) | 23 (41.8%) | 118 |  |
| Intrahepatic | 45 (28.7%) | 23 (26.1%) | 11 (20.5%) | 79 |  |
| Extrahepatic | 47 (29.9%) | 28 (31.8%) | 18 (32.7%) | 93 |  |
| *-hilar* | 28 | 16 | 8 | 52 |  |
| *-distal* | 19 | 12 | 10 | 41 |  |
| Ampulla of Vater | 3 (1.9%) | 4 (4.5%) | 3 (5.5%) | 10 |  |
| Biliary drainage |  |  |  |  | 0.037 |
| No | 81 (51.6%) | 56 (63.6%) | 38 (69.1%) | 175 |  |
| Yes | 76 (48.4%) | 32 (36.4%) | 17 (30.9%) | 125 |  |
| Prior primary resection |  |  |  |  | 0.173 |
| No | 126 (80.3%) | 64 (72.7%) | 38 (69.1%) | 228 |  |
| Yes | 31 (19.7%) | 24 (27.3%) | 17 (30.9%) | 72 |  |

ECOG PS, Eastern Cooperative Oncology Group performance status; GC, gemcitabine plus cisplatin; GS, gemcitabine plus S-1.
