# Supplementary material for: Assessment of heterogeneity according to hospital or medical experience factors in outcomes of chemotherapy for advanced biliary tract cancer: a post-hoc analysis of JCOG1113
Source: Jpn J Clin Oncol. 2025 Jan 8;55(4):355–61. doi: 10.1093/jjco/hyae188 (PMC11973634; doi:10.1093/jjco/hyae188)
Supplement: supple_Table_4_revise_hyae188 [file supple_table_4_revise_hyae188.docx]

|  | GC | | | | | | GS | | | | | | |
| --- | --- | --- | --- | --- | --- | --- | --- | --- | --- | --- | --- | --- | --- |
|  | **OS** | | | **PFS** | | | **OS** | | | **PFS** | | |  |
| Experience in medical oncology | Median  OS | Univariate HR  (95% CI)  [P-value] | Multivariate HR  (95% CI) [P-value] | Median PFS | Univariate HR  (95% CI) [P-value] | Multivariate HR  (95% CI) [P-value] | Median OS | Univariate HR  (95% CI) [P-value] | Multivariate HR  (95% CI) [P-value] | Median PFS | Univariate HR  (95% CI) [P-value] | Multivariate HR  (95% CI) [P-value] |  |
| Low | 14.1 | 1 | 1 | 6.9 | 1 | 1 | 15.1 | 1 | 1 | 6.9 | 1 | 1 |  |
| Intermediate | 10.7 | 0.854  (0.559-1.306) [0.467] | 0.884  (0.573-1.364) [0.578] | 6.7 | 1.170  (0.802-1.707) [0.414] | 1.246  (0.840-1.849) [0.275] | 16.0 | 1.180  (0.775-1.796) [0.441] | 1.165  (0.760-1.785) [0.483] | 6.2 | 1.149  (0.780-1.693) [0.481] | 1.107  (0.739-1.659) [0.621] |  |
| High | 5.0 | 1.409  (0.857-2.315) [0.176] | 1.515  (0.910-2.522) [0.110] | 4.5 | 1.538  (0.973-2.431) [0.065] | 1.613  (1.013-2.568) [0.044] | 18.5 | 0.948  (0.596-1.509) [0.823] | 1.040  (0.627-1.726) [0.879] | 8.5 | 0.983  (0.639-1.512) [0.937] | 0.990  (0.622-1.578) [0.967] |  |

Supplemental table 4. Comparison of OS/PFS among patients treated with either GC or GS from institutions classified into the highest, intermediate, and lowest tertiles by experience in medical oncology.

Multivariate analysis was adjusted for treatment group, sex distribution, age, distribution of ECOG PS, distribution of disease stage, distribution of primary site, and need for biliary drainage.
