# Supplementary material for: Assessment of heterogeneity according to hospital or medical experience factors in outcomes of chemotherapy for advanced biliary tract cancer: a post-hoc analysis of JCOG1113
Source: Jpn J Clin Oncol. 2025 Jan 8;55(4):355–61. doi: 10.1093/jjco/hyae188 (PMC11973634; doi:10.1093/jjco/hyae188)
Supplement: supple_Table_5_revise_hyae188 [file supple_table_5_revise_hyae188.docx]

Supplemental table 5**.** Patient characteristics in tertile groups divided by experience in biliary intervention.

|  | Lowest  （score;  197-337） | Intermediate  （score;  345-1238） | Highest  （score;  1395-3422） | Total | P value |
| --- | --- | --- | --- | --- | --- |
|  | N=37 | N=153 | N=110 | n=300 |  |
| Treatment |  |  |  |  | 0.754 |
| GC | 20 (54.1%) | 77 (50.3%) | 52 (47.3%) | 149 |  |
| GS | 17 (45.9%) | 76 (49.7%) | 58 (52.7%) | 151 |  |
| Age, years |  |  |  |  | 0.550 |
| Median | 67 | 67 | 69 | 67 |  |
| (Range) | (45-79) | (37-79) | (27-79) | (27-79) |  |
| Sex |  |  |  |  | 0.498 |
| Male | 24 (64.9%) | 83 (54.2%) | 63 (57.3%) | 170 |  |
| Female | 13 (35.1%) | 70 (45.8%) | 47 (42.7%) | 130 |  |
| ECOG PS |  |  |  |  | 0.076 |
| 0 | 24 (64.9%) | 101 (66.0%) | 86 (78.2%) | 211 |  |
| 1 | 13 (35.1%) | 52 (34.0%) | 24 (21.8%) | 89 |  |
| Disease stage |  |  |  |  | 0.676 |
| Localized | 7 (18.9%) | 25 (16.3%) | 20 (18.2%) | 52 |  |
| Metastatic | 18 (48.6%) | 94 (61.4%) | 64 (58.2%) | 176 |  |
| Recurrent | 12 (32.4%) | 34 (22.2%) | 26 (23.6%) | 72 |  |
| Primary site |  |  |  |  | 0.307 |
| Gallbladder | 18 (48.7%) | 59 (38.6%) | 41 (37.3%) | 118 |  |
| Intrahepatic | 9 (24.3%) | 43 (28.1%) | 27 (24.5%) | 79 |  |
| Extrahepatic | 8 (21.6%) | 44 (28.8%) | 41 (37.3%) | 93 |  |
| *-hilar* | 4 | 23 | 25 | 52 |  |
| *-distal* | 4 | 21 | 16 | 41 |  |
| Ampulla of Vater | 2 (5.4%) | 7 (4.6%) | 1 (0.9%) | 10 |  |
| Biliary drainage |  |  |  |  | 0.025 |
| No | 24 (64.9%) | 98 (64.1%) | 53 (48.2%) | 175 |  |
| Yes | 13 (35.1%) | 55 (35.9%) | 57 (51.8%) | 125 |  |
| Prior primary resection |  |  |  |  | 0.424 |
| No | 25 (67.6%) | 119 (77.8%) | 84 (76.4%) | 228 |  |
| Yes | 12 (32.4%) | 34 (22.2%) | 26 (23.6%) | 72 |  |

ECOG PS, Eastern Cooperative Oncology Group performance status; GC, gemcitabine plus cisplatin; GS, gemcitabine plus S-1.
